# Network analysis of cognition and function in Alzheimer’s disease: a cross-sectional study

**DOI:** 10.3389/fpsyt.2025.1661313

**Published:** 2025-11-21

**Authors:** Linshuang Hu, Yun Chen, Lili Chen, Xinwu Ye, Yefu Wang, Liandan Huang, Ying Wang, Xianghe Zhu, Sunke Chen

**Affiliations:** 1 Department of Psychotherapy, Wenzhou Seventh People’s Hospital, Wenzhou, China; 2 Department of Geriatric, Wenzhou Geriatric Hospital, Wenzhou, China; 3 The Affiliated Kangning Hospital of Wenzhou Medical University, Zhejiang Provincial Clinical Research Center for Mental Health, Wenzhou, China; 4 School of Mental Health, the Affiliated Kangning Hospital, Zhejiang Provincial Clinical Research Center for Mental Health, Wenzhou Medical University, Wenzhou, Zhejiang, China

**Keywords:** Alzheimer, network analysis, cognition, function, regularized

## Abstract

**Objectives:**

Traditional approaches in Alzheimer’s disease (AD) research examine cognitive symptoms in isolation, potentially overlooking dynamic interrelationships among impairment domains. This study employed network analysis to examine structural organization of cognitive and functional domains in mild (mAD) and moderate-to-severe (Mod-sAD) Alzheimer’s disease, aiming to identify stage-specific symptom structures and inform targeted interventions.

**Methods:**

A cross-sectional study included 134 participants diagnosed with AD according to DSM-5 criteria. Participants were classified into mAD (n=37) and Mod-sAD (n=97) groups. Regularized partial correlation networks with extended Bayesian information criterion regularization examined symptom interdependencies across six CDR domains: memory, orientation, judgment and problem solving, community affairs, home and hobbies, and personal care. Network comparison tests and centrality analyses identified structural differences between disease stages.

**Results:**

The Mod-sAD group demonstrated significantly higher impairment scores across all domains (*p* < 0.001) with large effect sizes (Cohen’s *d*: 1.83-2.71). Network analysis revealed increased global strength in Mod-sAD versus mAD networks (2.60 vs. 2.49, *p* < 0.05), indicating greater symptom interconnectedness in advanced stages. Centrality analyses revealed fundamental reorganization: memory emerged as most central in Mod-sAD (*strength* = 1.62), while judgment and problem-solving showed highest centrality in mAD (*strength* = 1.65). Orientation centrality increased substantially across progression (*strength*: -1.32 to 0.40).

**Conclusions:**

AD progression features increasing network density and centrality shifts from executive-function-centered networks in mild AD to memory-centered networks in moderate-to-severe stages. Findings suggest stage-specific interventions: executive enhancement in mild AD and memory-focused approaches in advanced stages.

## 1 Introduction

Alzheimer’s disease (AD) is the leading cause of dementia worldwide, accounting for 60–80% of all cases (1). Driven by global population aging, the prevalence of AD is rising rapidly, posing significant medical, social, and economic challenges (2). Currently, more than 55 million individuals worldwide are living with dementia, a figure projected to surpass 139 million by 2050 (3). In China, demographic transitions have further intensified this issue: in 2020, 13.5% of the population was aged ≥65 years, and this proportion is expected to exceed 30% by 2050. Concurrently, the number of individuals with dementia is projected to triple—from 16.3 million in 2020 to 49 million by 2050—placing immense pressure on the healthcare system (4). Clinically, AD is characterized as a progressive neurodegenerative disorder, with symptoms evolving over time and exhibiting distinct features at different stages (5). The core trajectory of this progression is reflected primarily in two domains—cognition and function—which together capture the decline in mental processes and the resulting loss of independence. Cognition can be understood as a neural process underlying information acquisition and processing, whereas function reflects the capacity to maintain independence in everyday life. Although distinct, these domains are intrinsically linked, with cognitive decline consistently shown to be associated with functional impairment (6). The early or mild stage (mAD) is typically marked by subtle memory deficits and mild impairments in complex instrumental activities of daily living, such as financial management and social participation (6). As the disease advances to the moderate-to-severe stage (Mod-sAD), patients experience severe impairments in memory, orientation, and personal care, ultimately resulting in near-complete dependence on others for daily functioning (6, 7). This stage-dependent trajectory underscores the critical importance of early detection and stage-specific interventions to delay cognitive deterioration and preserve functional abilities (8, 9). However, in China, delayed diagnosis of early-stage or mild dementia remains a major challenge, often resulting in missed opportunities for effective management and intervention. Recent evidence indicates that the average time from symptom onset to formal diagnosis exceeds two years, and fewer than 30% of individuals with dementia are diagnosed in a timely manner (10). These delays often result in missed opportunities for early intervention, when treatment may be most effective. For instance, Rasmussen et al. (2019) demonstrated that interventions tailored to patients’ cognitive profiles in the early stages can significantly delay functional deterioration and improve overall quality of life (11). Understanding the evolution of cognitive and functional features across disease stages is essential for optimizing individualized treatment and care strategies, as well as informing policy development and resource allocation.

Given these stage-specific challenges in characterizing AD, there is a pressing need for analytical approaches that move beyond isolated symptom assessment and instead capture the dynamic interrelationships among cognitive and functional domains. Traditional analytical approaches in dementia research often rely on latent variable models, which posit that observable symptoms are manifestations of an underlying, unobservable disease construct. While this framework has utility in various contexts, it obscure direct and dynamic interactions among symptoms. In contrast, network analysis offers a fundamentally different conceptual and methodological approach (12). Rather than viewing symptoms as passive indicators of a latent condition, it treats them as active, interacting elements within a network (10, 13). This allows for the identification of core or central symptoms—those most strongly connected to others—that may play a pivotal role in maintaining or propagating overall dysfunction (11, 14). For instance, studies on depression have shown that symptoms with high centrality measures, such as depressed mood and anhedonia, act as critical bridges connecting other symptom clusters; targeting these central symptoms leads to more effective treatment outcomes (15). Similarly, research on mild cognitive impairment has revealed that memory-related symptoms often emerge as central nodes influencing the activation of other cognitive domains, suggesting that early interventions targeting memory networks may help prevent cascading cognitive decline (16). In the context of AD, examining the symptom networks differ across stages of disease progression can provide valuable insights into core symptom dynamics and inform the development of stage-specific therapeutic strategies.

The present study aims to examine the network structures of cognitive and functional in patients with mAD and Mod-sAD. Specifically, we seek to identify changes in core symptom profiles by comparing cognitive features across disease stages through network analytical methods. We hypothesize that the Mod-sAD group will exhibit a denser and more interconnected symptom network, with shifts in centrality reflecting changes in dominant symptom patterns. By elucidating stage-specific symptom structures, this study aims to provide a more nuanced understanding of AD progression and inform the development of stage-adapted clinical interventions.

## 2 Method

### 2.1 Participants

Participants were recruited from the Psychiatric Hospital of Wenzhou between January 2024 and January 2025. All participants were diagnosed with Alzheimer’s disease according to the criteria of the Diagnostic and Statistical Manual of Mental Disorders, Fifth Edition (DSM-5) (17). The diagnostic process included comprehensive clinical evaluations conducted by two licensed psychiatrists, with at least one holding a senior professional title, to ensure diagnostic accuracy and reliability. To minimize diagnostic bias, a consensus diagnosis was required in cases of initial disagreement between clinicians. Inclusion criteria were as follows: (1) age 60 years or older; (2) confirmed diagnosis of Alzheimer’s disease based on DSM-5 criteria; (3) availability of complete Clinical Dementia Rating (CDR) assessments; and (4) provision of written informed consent by participants or their legally authorized representatives. Exclusion criteria included: (1)history of stroke with focal neurological signs and imaging findings consistent with cerebral small vessel disease (Fazekas score ≥2); (2) mental or intellectual developmental disorders; (3) other diseases known to cause cognitive impairment; (4) comorbid conditions that would prevent cooperation with cognitive assessments; (5) refusal to provide informed consent; (6) insufficient clinical documentation in the case report form; (7) focal neurological symptoms and signs consistent with stroke (including hemiplegia, central facial paralysis, Babinski’s sign, sensory disturbances, dysarthria); (8) CT/MRI evidence of multiple macrovascular infarctions, lacunar infarctions, extensive periventricular white matter lesions, or strategically located single infarcts; and (9) clinical diagnosis of vascular dementia. A total of 134 participants met the inclusion criteria and were included in the final analysis.

### 2.2 Procedure

All participants underwent individual, face-to-face clinical assessments conducted in the outpatient clinic of the hospital. Each evaluation was performed one-on-one by a physician holding a valid psychiatric practice registration certificate. The assessment protocol included structured clinical interviews, during which demographic information, medical history, and cognitive assessments were systematically collected. The Clinical Dementia Rating (CDR) scale was administered by trained clinicians, who rated participants across six functional domains: memory, orientation, judgment and problem solving, community affairs, home and hobbies, and personal care. Each assessment session lasted approximately 45–60 minutes to ensure a thorough evaluation of all domains. Participants were subsequently classified into two groups based on their CDR global scores: the mAD group, with CDR scores of 0.5–1.0, and the Mod-sAD group, with CDR scores of 2.0–3.0 (18).

### 2.3 Measurements

#### 2.3.1 Demographic information

Demographic data was collected through structured interviews with participants and their caregivers during clinical assessments. The collected information included age, gender, educational level, smoking status, and drinking status. Age was recorded in years as a continuous variable. Gender was classified as male or female. Educational level was categorized into three groups: primary school or below, junior high school, and senior high school or above. Smoking and drinking statuses were each classified dichotomously as “yes” or “no” based on self-report.

#### 2.3.2 CDR assessment

Cognitive function was assessed using the Clinical Dementia Rating (CDR) scale, a widely validated instrument for staging dementia severity. The CDR evaluates cognitive and functional performance across six domains: memory, orientation, judgment and problem solving, community affairs, home and hobbies, and personal care. Each domain is rated on a 5-point scale. Memory domain assessment focused on recent and remote memory for events, facts, and personal information. Orientation evaluation examined awareness of time, place, and person. Judgment and problem solving assessed the participant’s ability to handle complex situations and make reasonable decisions. Community affairs evaluation examined the participant’s ability to function independently outside the home, including shopping, managing finances, and engaging in social activities. Home and hobbies assessment focused on domestic responsibilities, leisure activities, and intellectual interests. Personal care evaluation assessed the participant’s ability to maintain personal hygiene and self-care activities. The CDR assessment was conducted through semi-structured interviews with both participants and their informants (typically family members or primary caregivers).

## 3 Statistical analysis

All statistical analyses were performed using R software (version 4.3.1). Descriptive statistics were used to summarize the demographic and clinical characteristics of the participants. Continuous variables were expressed as means ± standard deviations, and categorical variables were presented as frequencies and percentages. Group comparisons between mAD and Mod-sAD were conducted using independent sample t-tests for continuous variables and chi-square tests for categorical variables. When the expected cell counts were small, Fisher’s exact test was applied. Pearson correlation analyses were conducted to explore associations between demographic variables and CDR domain scores.

Symptom networks were constructed using regularized partial correlation networks through the R package “qgraph” (19). Regularization was applied using the Extended Bayesian Information Criterion (EBIC) with a hyperparameter γ = 0.5 to balance model complexity and sparsity. Separate networks were estimated for the mAD and Mod-sAD groups to examine structural differences between disease stages. Network comparison was performed using the Network Comparison Test (NCT) implemented in the “NetworkComparisonTest” package (20). Global strength differences between networks were tested using permutation-based methods with 1000 iterations. Network subtraction was employed to identify edges that differed between groups, highlighting connections that emerged or strengthened in the moderate-to-severe stage. Centrality indices were calculated for each node within both networks, including strength centrality (sum of absolute edge weights connected to a node), closeness centrality (inverse of the sum of shortest path lengths to all other nodes), and betweenness centrality (frequency with which a node lies on the shortest path between other nodes). Centrality measures were standardized (z-scores) to facilitate comparison between groups and identify nodes with particularly high or low centrality values.

## 4 Result

### 4.1 Descriptive analytics

A total of 134 participants diagnosed with Alzheimer’s disease were included in the final analysis, comprising 37 patients with mAD and 97 patients with Mod-sAD. The demographic characteristics of the participants were presented in **Table 1**. The two groups were well-matched across most demographic variables, with no significant differences observed in gender distribution (*p* = 0.11), educational levels (*p* = 0.75), smoking status (*p* = 0.36), or drinking habits (*p* = 0.22). However, patients in the Mod-sAD group were significantly older than those in the mAD group (*p* = 0.009).

**Table 1:** Demographic characteristics of study participants.

Variables	mAD *n=37*	Mod-sAD *n=97*	*χ^2^/t*	*p*
Gender			2.54	0.11
Female	19 (22.35%)	66 (77.65%)		
Male	18 (36.73%)	31 (63.27%)		
Age (years)	71.62 ± 5.22	76.14 ± 9.85	2.65	0.009
Educational levels			0.58	0.75
Primary School or Below	21 (26.58%)	58 (73.42%)		
Junior High School	11 (32.35%)	23 (67.65%)		
Senior High School or Above	5 (23.81%)	16 (76.19%)		
Smoking			(–)^a^	0.36
No	34 (26.56%)	94 (73.44%)		
Yes	3 (50.00%)	3 (50.00%)		
Drinking			(–)^a^	0.22
No	33 (26.19%)	93 (73.81%)		
Yes	4 (50.00%)	4 (50.00%)		

a Fisher’s exact test was used due to small, expected cell frequencies; mAD, mild Alzheimer’s disease; Mod-Sad, moderate-to-severe Alzheimer’s disease.

### 4.2 Cognitive and functional differences at different stages

**Figure 1:**
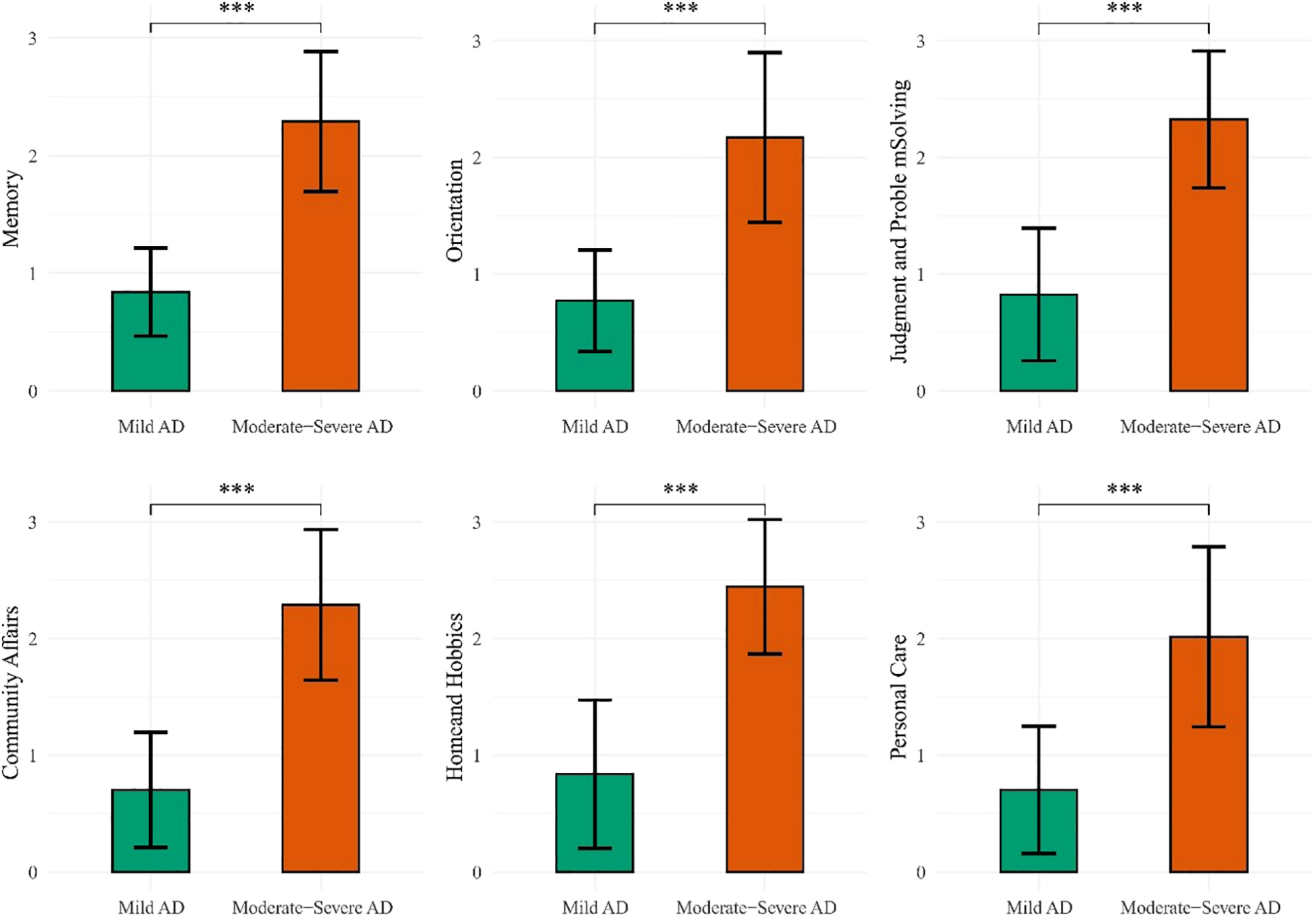
Clinical dementia rating comparison between groups. ***p < 0.001.

Significant differences were observed across all six domains of the CDR between the mAD and mod-sAD groups (**Figure 1**). The Mod-sAD group showed significantly higher impairment scores, compared to the mAD group, across all cognitive and functional domains (all *p* < 0.001). Cohen’s *d* values indicated large effect sizes across all domains, with Home and Hobbies showing the largest effect size (Cohen’s *d* = 2.71), followed by Memory (Cohen’s *d* = 2.67), Community Affairs (Cohen’s *d* = 2.61), and Judgment and Problem Solving (Cohen’s *d* = 2.58). Orientation (Cohen’s *d* = 2.12) and Personal Care (Cohen’s *d* = 1.83). Correlation analysis revealed significant relationship between demographic variables and CDR domain scores (presented in **Table 2**). Gender was negatively correlated with memory impairment (*r* = –0.18, *p* < 0.05). Age was positively correlated with all functional domains, including memory (*r* = 0.22, *p* < 0.05), orientation (*r* = 0.19, *p* < 0.05), judgment and problem solving (*r* = 0.18, *p* < 0.05), community affairs (*r* = 0.22, *p* < 0.01), home and hobbies (r = 0.33, *p* < 0.001), and personal care (*r* = 0.33, *p* < 0.001).

**Table 2:** Correlation matrix of demographic characteristics and CDR (*n* = 134).

Variables	1.	2.	3.	4.	5.	6.	7.	8.	9.	10.	
1.Gender	1										
2.Age	-0.13	1									
3.Educational levels	0.25^**^	0.09	1								
4.Smoking	0.29^***^	-0.14	-0.03	1							
5.Drinking	0.14	-0.14	0.12	0.55^***^	1						
6.Memory	-0.18^*^	0.22^*^	0.02	-0.08	-0.12	1					
7.Orientation	-0.13	0.19^*^	0.01	-0.05	-0.13	0.88^***^	1				
8.Judgment and problem solving	-0.07	0.18^*^	0.03	-0.08	-0.10	0.88^***^	0.82^***^	1			
9.Community affairs	-0.04	0.22^**^	0.10	-0.14	-0.18^*^	0.81^***^	0.78^***^	0.84^***^	1		
10.Home and hobbies	-0.06	0.33^***^	-0.01	-0.10	-0.15	0.84^***^	0.78^***^	0.86^***^	0.87^***^	1	
11.Personal care	-0.11	0.33^***^	0.07	-0.07	-0.13	0.79^***^	0.78^***^	0.81^***^	0.81^***^	0.82^***^	1

*p < 0.05; **p < 0.01; ***p < 0.001.

### 4.3 Network comparison and across Alzheimer’s disease sages

**Figure 2:**
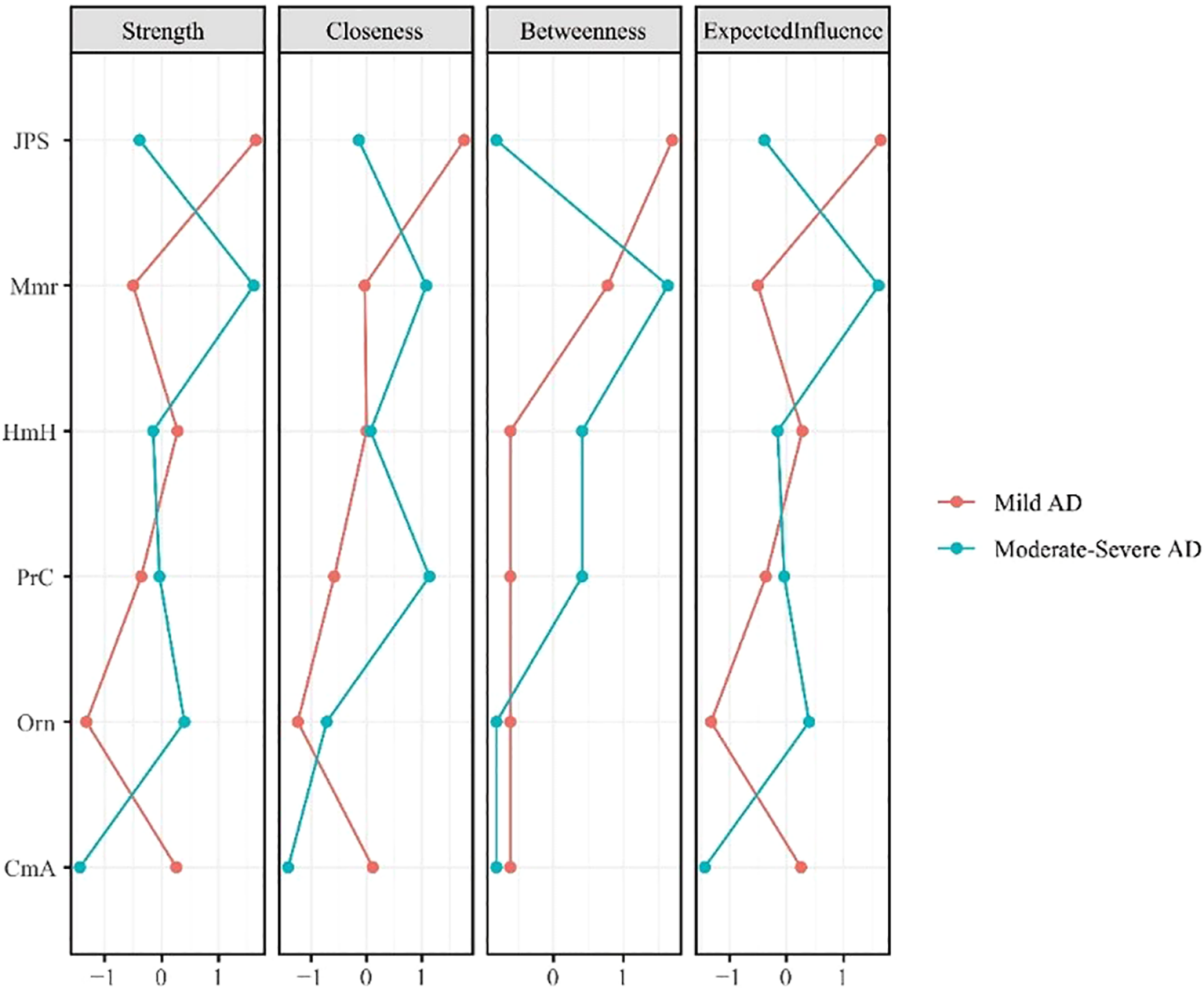
Centrality indices (strength, closeness, betweenness, expected influence) for mild and moderate-severe Alzheimer’s disease symptom networks.

**Figure 3:**
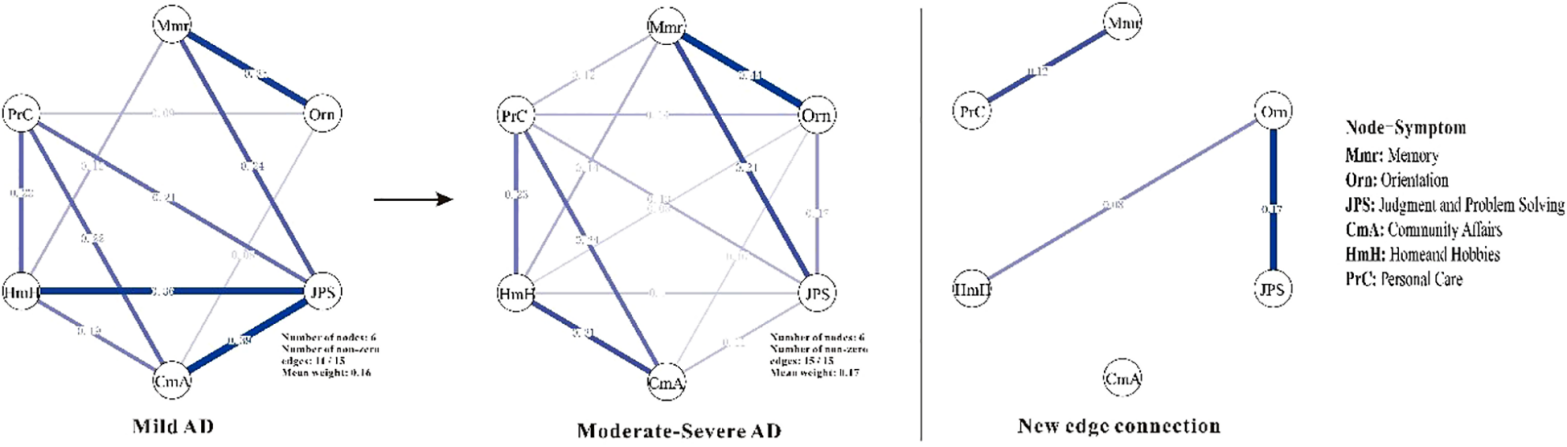
Symptom networks in mild and moderate-severe Alzheimer’s disease groups, and the difference network. Mmr, Memory; Orn, Orientation; JPS, Judgment and Problem Solving; CmA, Community Affairs; HmH, Home and Hobbies; PrC, Personal Care. Line thickness reflects the strength of the regularized partial correlations between nodes—thicker lines indicate stronger associations. Edge color denotes the direction of the association, with positive correlations shown in blue.

Centrality analyses (**Figure 2**) further highlights shifts in node importance, particularly in terms of strength. In the mAD network (**Figure 3**), judgment and problem-solving exhibited the highest strength (*strength* = 1.65). By contrast, in the Mod-sAD network, memory emerged as the node with the highest strength (*strength* = 1.62), while the strength of judgment and problem-solving showed a sharp decline (*strength* = −0.39). Moreover, the Mod-sAD network demonstrated increased strength values for orientation (*strength* = 0.40) relative to the mAD group (*strength* = −1.32). In addition, memory showed a notable increase in both closeness (*closeness* = 1.08) and betweenness (*betweenness* = 1.63), compared to the mAD group (*closeness* = −0.04; *betweenness* = 0.78). Considering that all edge weights were positive, expected influence values were identical to strength values and thus were not reported separately.

### 4.4 Clustering coefficient analysis

**Figure 4:**
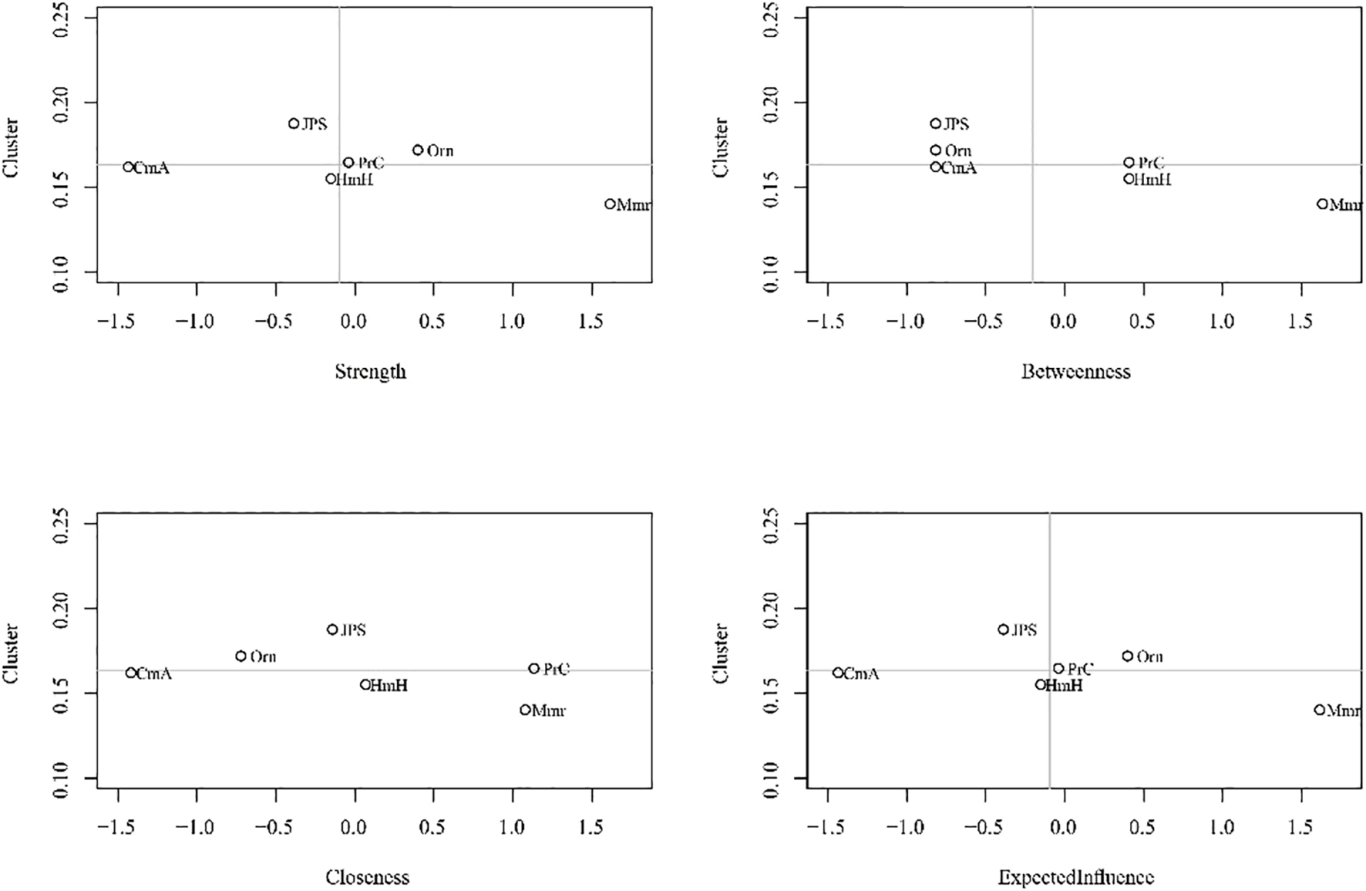
Clustering coefficients and centrality measures of the symptom network.

To assess potential redundancy among the CDR domains and to rule out clustering coefficient inflation that might bias centrality estimates, clustering coefficients were calculated for each node. Across all nodes, clustering coefficients were relatively low, ranging from 0.11 to 0.18, with an overall mean clustering coefficient of 0.16 (*SD* = 0.02). The node with the highest clustering coefficient was judgment and problem solving (Clustering coefficient = 0.18). As shown in **Figure 4**, there was no significant correlation between strength and clustering coefficient in the symptom network model(|*r*| < 0.30, *p* > 0.56).

## 5 Discussion

This study utilized network analysis to examine the network structure based on CDR domains based on CDR domains in individuals with mild and moderate-to-severe Alzheimer’s disease, offering novel insights into the dynamic interplay of symptoms across the course of disease progression. The findings reveal alterations in symptom network structures that closely parallel the clinical trajectory of AD as it advances from mild to more severe stages.

### 5.1 Network density and global connectivity

One of the most salient findings of this study was the significantly greater network density and global strength observed in the moderate-to-severe Alzheimer’s disease (Mod-sAD) group compared to the mild AD (mAD) group. This pattern suggests that individuals with more severe AD tend to show more interconnected cognitive and functional impairments, reflecting a more integrated system of deficits. These results are consistent with the cascade hypothesis of neurodegeneration, which posits that pathological processes in AD propagate through interconnected brain networks, resulting in progressive deterioration across multiple cognitive domains (21, 22). The increased connectivity observed in advanced stages may be indicative of more pervasive and interdependent functional impairments. Although our data, based solely on CDR domains, cannot directly address underlying neuropathological mechanisms, this pattern is broadly in line with neuroimaging evidence showing altered connectivity in Alzheimer’s disease (23, 24). For example, Brier et al. (2014) reported that disruptions in default mode network connectivity are associated with greater cognitive impairment severity, suggesting that network-level alterations may contribute to the clinical manifestations of advanced AD (25).

### 5.2 Centrality shifts and symptom dynamics

The centrality analyses revealed a fundamental reorganization of core symptoms between disease stages, with memory becoming the node with the highest strength and betweenness in moderate to severe AD. This finding provides important insights into the evolving role of memory dysfunction throughout AD progression. In the early stages, memory impairments represented a relatively isolated deficit that has not yet extensively permeated other cognitive domains. Across disease stages, memory dysfunction appears increasingly central within the overall symptom profile, suggesting that memory-related deficits may be linked to a broader pattern of functional impairments. This shift in centrality aligns with the well-established understanding of AD as primarily a disorder of memory systems, particularly involving the hippocampus and associated medial temporal lobe structures (26, 27). The increased centrality of memory in moderate-to-severe stages reflects the progressive involvement of memory-related neural networks, which become increasingly critical for maintaining other cognitive functions. Greater impairment of memory systems may coincide with widespread dysfunction across interconnected cognitive domains. Conversely, judgment and problem-solving demonstrated the highest centrality in mild AD but showed a dramatic decline as the disease progressed to moderate-to-severe stages.

The substantial increase in orientation centrality from mild to moderate-to-severe AD represents another significant finding warranting discussion. Orientation, encompassing awareness of time, place, and person, showed markedly increased connectivity and influence within the symptom network as AD progressed. This finding may reflect the fundamental role of orientation in organizing and coordinating other cognitive functions (28). As orientation becomes increasingly impaired in advanced AD, it may serve as a critical bridge connecting various cognitive deficits, potentially explaining why disorientation is such a prominent and distressing feature of moderate-to-severe dementia. Gennaro et al. further highlighted that the conversion from normal aging to AD may be traced through allocentric distance-based deficits, a core component of spatial orientation capacity, underscoring the pivotal role of orientation impairments in early disease detection and progression monitoring (28). From a clinical perspective, the emergence of orientation as a central node in advanced AD has important implications for assessment and intervention strategies.

### 5.3 Newly network connections in AD progression

Beyond the centrality symptoms shifts, several new edge connections emerged as AD progressed from mild to moderate-to-severe stages. Specifically, Memory-Personal Care, Orientation-Judgment and Problem Solving, and Orientation-Home and Hobbies—provides insights into the dynamic reorganization of cognitive-functional networks during disease progression. This finding aligns with previous network studies suggesting that AD progression involves not merely the loss of connections, but also the formation of new pathological or compensatory pathways (29). The Memory-Personal Care connection particularly supports the cascade model of functional decline, where cognitive impairments progressively impact instrumental and basic activities of daily living (30, 31). This coupling reflect the increasing reliance of self-care abilities on intact memory systems, consistent with studies showing that memory deficits predict functional deterioration in moderate-stage AD (32). The emergence of Orientation-Judgment and Problem-Solving connections corroborate findings that executive dysfunction and disorientation become increasingly interrelated as AD advances (33, 34). Similarly, the Orientation-Home and Hobbies connection reflects the documented relationship between spatial disorientation and the abandonment of complex leisure activities (35). These emerging connections represent either compensatory recruitment of cognitive resources, as suggested by neuroimaging studies showing hyperactivation in early AD stages (36), or pathological coupling reflecting shared vulnerability to neurodegeneration (37). Future longitudinal network studies are needed to distinguish between these mechanisms and their implications for intervention strategies.

## 6 Implications for stage-specific interventions

The present findings provide valuable insights for developing stage-adapted clinical interventions in Alzheimer’s disease. The identification of memory as the most central node in moderate-to-severe AD underscores the importance of prioritizing memory-focused interventions at this stage. Therapeutic approaches such as cognitive stimulation therapy, memory-specific rehabilitation strategies, and pharmacological treatments targeting cholinergic deficits may be particularly beneficial for attenuating global functional decline in advanced stages. Furthermore, the increased centrality of orientation highlights the potential utility of incorporating orientation-supportive interventions, including reality orientation therapy and environmental modifications, to mitigate disorientation-related distress and improve overall daily functioning (38). In mild AD, interventions should focus on supporting judgment and problem-solving capacities, which emerged as the most central in this stage. Targeting executive functions early through problem-solving training and compensatory strategies could delay further cognitive deterioration and maintain independence longer (39).

## 7 Limitation

Several limitations of this study should be acknowledged. First, the cross-sectional design precludes causal inferences about the dynamic evolution of symptom networks over time. Although we identified distinct network structures and centrality patterns between mild and moderate-to-severe Alzheimer’s disease, these reflect group-level comparisons and not within-person changes. Future longitudinal studies are necessary to determine whether these observed differences represent true progression dynamics, particularly during the transition from mild to advanced stages. Second, although the Clinical Dementia Rating scale is widely validated and offers comprehensive coverage of cognitive and functional domains, it may not capture more subtle neuropsychiatric symptoms that also influence network dynamics. Future research integrating neuropsychiatric symptom assessments (e.g., Neuropsychiatric Inventory) could provide a more holistic understanding of symptom interrelations. Third, the sample size, while adequate for exploration network analysis, limits generalizability. As the sample was drawn exclusively from a Chinese population, the generalizability of our findings to other ethnic or cultural groups may be limited. Cultural factors can influence how cognitive and functional symptoms are expressed, perceived, and reported, potentially affecting network structures. While this limitation cannot be addressed within the scope of the present study, future cross-cultural research is needed to validate and extend these findings in more diverse populations.

## 8 Conclusion

This study provides quantitative evidence of stage-specific differences in the network structure of cognitive and functional symptoms in Alzheimer’s disease. Our findings revealed significantly greater impairments across all CDR domains in the Mod-sAD group compared to the mAD group, with large effect sizes. Network analysis further demonstrated increased global connectivity in the Mod-sAD group, indicating higher interdependence among symptoms as the disease progresses. Centrality analyses identified a shift from judgment and problem-solving as the most central domain in mild AD to memory in moderate-to-severe AD, along with increased centrality of orientation. These results suggest that symptom structures in AD evolves with disease severity, underscoring the importance of stage-specific assessment and intervention strategies. These findings provide a foundation for future research into targeted, network-informed approaches to dementia care.

## Data Availability

The raw data supporting the conclusions of this article will be made available by the authors, without undue reservation.
